# Supplementary material for: The Systems Biology Research Tool: evolvable open-source software
Source: BMC Syst Biol. 2008 Jun 29;2:55. doi: 10.1186/1752-0509-2-55 (PMC2446383; doi:10.1186/1752-0509-2-55)
Supplement: Additional file 1 — SBRT Archive. An archive of the current version of the Systems Biology Research Tool. [file 1752-0509-2-55-S1.zip › sbrt-1.4.0/doc/developers_guide/api/sbrt/kernel/math/stat/NormalDist.html]

NormalDist


|  |  |  |
| --- | --- | --- |
| **Overview** \| **Package** \| **Class** \| **Use** \| **Tree** \| **Deprecated** \| **Index** \| **Help** | |  |
| PREV CLASS   NEXT CLASS | **FRAMES**    **NO FRAMES**     **All Classes** |  |
| SUMMARY: NESTED \| FIELD \| CONSTR \| METHOD | DETAIL: FIELD \| CONSTR \| METHOD |  |


---


## sbrt.kernel.math.stat Class NormalDist

```
java.lang.Object
  sbrt.kernel.math.stat.NormalDist
```

---

``` class NormalDist extends java.lang.Object ```

This class is used to represent normal distributions.

**Author:**
:   This class was written and documented by Jeremiah Wright while
    in the Wagner lab.

---

**Constructor Summary**

`NormalDist(double average,
double stdev)`
  
          Constructs a new normal distribution with the provided average
and standard deviation.


| **Method Summary** | |
| --- | --- |
| `double` | `getMean()`             Returns the mean, or average, of this distribution. |
| `double` | `getStdev()`             Returns the standard deviation of this distribution. |
| `double` | `getVariance()`             Returns the variance of this distribution. |

**Methods inherited from class java.lang.Object**

`clone, equals, finalize, getClass, hashCode, notify, notifyAll, toString, wait, wait, wait`

**Constructor Detail**

### NormalDist

```
public NormalDist(double average,
                  double stdev)
```

:   Constructs a new normal distribution with the provided average
    and standard deviation.

    **Parameters:**: `average` - the mean of the distribution.: `stdev` - the standard deviation of the distribution.


**Method Detail**

### getMean

```
public double getMean()
```

:   Returns the mean, or average, of this distribution.

    :   **Returns:**: the mean, or average, of this distribution.

---


### getStdev

```
public double getStdev()
```

:   Returns the standard deviation of this distribution.

    :   **Returns:**: the standard deviation of this distribution.

---


### getVariance

```
public double getVariance()
```

:   Returns the variance of this distribution.

    :   **Returns:**: the variance of this distribution.


---


|  |  |  |
| --- | --- | --- |
| **Overview** \| **Package** \| **Class** \| **Use** \| **Tree** \| **Deprecated** \| **Index** \| **Help** | |  |
| PREV CLASS   NEXT CLASS | **FRAMES**    **NO FRAMES**     **All Classes** |  |
| SUMMARY: NESTED \| FIELD \| CONSTR \| METHOD | DETAIL: FIELD \| CONSTR \| METHOD |  |


---
